# Fatal hemolytic anemia associated with metformin: A case report

**DOI:** 10.1186/1752-1947-2-300

**Published:** 2008-09-10

**Authors:** Clifford D Packer, Thomas R Hornick, Sarah A Augustine

**Affiliations:** 1 Louis Stokes Cleveland Veterans Affairs Medical Center, East Boulevard, Cleveland, OH 44106, USA; 2 Case Western Reserve University School of Medicine, 10900 Euclid Avenue, Cleveland, OH 44106, USA

## Abstract

**Introduction:**

Metformin is a widely prescribed biguanide antidiabetic drug that has been implicated as a cause of hemolytic anemia in three previous case reports. We report a case of rapidly fatal hemolysis that was temporally associated with the initiation of metformin treatment for diabetes. Clinicians need to be aware of this rare but potentially serious side effect of metformin.

**Case presentation:**

A 56-year-old Caucasian man with type 2 diabetes mellitus was started on metformin to improve glycemic control. Shortly afterwards, he developed progressive fatigue, exertional dyspnea, cranberry-colored urine and jaundice. Laboratory studies showed severe hemolysis, with a drop in hemoglobin from 14.7 to 6.6 g/dl over 4 days, markedly elevated lactate dehydrogenase, bilirubin and reticulocyte counts, and a low haptoglobin level. A peripheral blood smear showed no schistocytes, and a direct Coombs test was positive for anti-IgG and negative for anti-C3. Despite corticosteroid treatment and transfusion of packed red blood cells, the patient developed increasing dyspnea, hypotension, further decline in hemoglobin to 3.3 g/dl, and fatal cardiorespiratory arrest 12 hours after admission.

**Conclusion:**

The serologic findings in this case suggest an autoimmune hemolytic anemia, caused either by a drug-induced autoantibody or a warm autoantibody. Based on the temporal association with metformin and the lack of other clear precipitating causes, we propose that metformin-induced hemolysis with a drug-induced autoantibody is a strong possibility. This mechanism differs from a previously described case with a possible antibody to the erythrocyte-drug complex. It has been shown, however, that hemolysis may occur via multiple mechanisms from the same drug. Clinicians should consider the possibility of metformin-associated immune hemolytic anemia in patients with otherwise unexplained hemolysis.

## Introduction

Metformin-induced hemolytic anemia has been reported in three patients, all of whom recovered when metformin was discontinued [1-3]. We report the case of a patient with fulminant and fatal hemolysis that occurred shortly after metformin was started for the treatment of type 2 diabetes mellitus.

## Case presentation

A 56-year-old Caucasian man with type 2 diabetes mellitus, hypothyroidism, idiopathic thrombocytopenic purpura status post splenectomy in 1979, and remote testicular cancer status post orchiectomy and radiation therapy in the 1970s was admitted with 2 days of progressive fatigue, exertional dyspnea, low back pain and cranberry-colored urine. He had been started on metformin 500 mg twice a day 4 days previously for type 2 diabetes but called in to his physician the following day with complaints of "palpitation, heavy breathing, and tossing and turning last night" after taking the metformin, which he declined to continue. Two days before admission he was seen at an outside emergency room with hematuria and anemia; he was diagnosed with a urinary tract infection and started on oral ciprofloxacin. His other medications were pantoprazole, levothyroxine and glyburide. On the day of admission, vital signs were temperature 37.1°C, pulse rate of 133 per minute, respiration rate of 32 breaths per minute, blood pressure of 155/84 and pulse oxygen 94% on 6 liters O_2_. Physical examination was significant for respiratory distress, scleral icterus and generalized jaundice. There was no jugular venous distention and no enlarged thyroid gland or lymphadenopathy. His lungs were clear, heart sounds were distant, his abdomen was soft without masses or enlargement of organs, he had no rashes and no peripheral edema. Mild lumbar and thoracic spinous tenderness was noted; neurological examination revealed mild lethargy but otherwise normal mental status, with no focal findings. Laboratory studies (Table 1) were significant for hemoglobin of 6.6 g/dl, which had dropped from 14.7 g/dl 4 days before, total bilirubin 6.6 mg/dl (direct 2.7 mg/dl), reticulocyte count 3.51%, lactate dehydrogenase 4829 U/l, and haptoglobin less than 6 mg/dl, all consistent with severe hemolysis. The direct antiglobulin (Coombs) test (DAT) was positive for anti-IgG and negative for anti-C3. The peripheral blood smear on admission significantly showed no schistocytes; 4% immature granulocytes and 4% nucleated red blood cells (RBCs) were noted. Other significant findings included marked leukocytosis to 46.1 K/cmm, acute renal failure, elevation of troponin-I and marked transaminase elevations (aspartate aminotransferase (AST) 1711, alanine aminotransferase (ALT) 806) with normal international normalized ratio and alkaline phosphatase. Blood and urine cultures showed no growth. Despite treatment with corticosteroids and transfusion of packed RBCs, the patient became increasingly dyspneic and agitated, complained of abdominal pain and developed hypotension followed by cardiorespiratory arrest approximately 8 hours after admission. Over the next 4 hours he was treated following the advanced cardiac life support protocol for recurrent bouts of pulseless electrical activity and asystole, with worsening hyperkalemia and metabolic acidosis. Despite transfusion, hemoglobin was noted to have declined to 3.3 g/dl. The patient died approximately 12 hours after admission.

**Table 1:** Laboratory test results

Variable	11/21/2006	11/20/2006	11/16/2006	9/21/2006	Units
Hemoglobin	3.3	6.6	14.7	-	g/dl
Hematocrit	10.2	19.5	43.3	-	%
Platelet	97	166	322	-	K/cmm
White blood cell	44.0	46.1	11.5	-	K/cmm
Glucose	-	277	-	220	mg/dl
Blood urea nitrogen	-	38	-	22	mg/dl
Creatinine	-	2.4	-	1.1	mg/dl
Sodium	-	129	-	140	meq/l
Potassium	-	5.5	-	4.5	meq/l
Chloride	-	94	-	104	meq/l
CO_2_	-	15	-	27	mmol/l
Calcium	-	8.7	-	9.2	mg/dl
Lactate dehydrogenase	-	4829	-	-	U/l
Haptoglobin	-	<6	-	-	mg/dl
Fibrinogen	-	253	-	-	mg/dl
Total bilirubin	-	6.9	-	0.8	mg/dl
Direct bilirubin	-	-	-	0.1	mg/dl
Alkaline phosphatase	-	103	-	83	U/l
Alanine aminotransferase	-	806	-	38	U/l
Aspartate aminotransferase	-	1711	-	31	U/l
Creatine phosphokinase	-	597	-	-	U/l
Partial thromboplastin time	-	26.7	-	-	seconds
International normalized ratio	-	1.22	-	-	-

## Discussion

The temporal relationship with metformin ingestion is strong in this case, with the patient noting symptoms within hours of the initiation of metformin. The 4-day course was rapid and fatal, characterized by massive hemolysis and shock. In the three previously reported cases of metformin-induced hemolytic anemia (Table 2), the time to onset of symptoms ranged from 9 to 14 days after starting metformin, and none resulted in massive hemolysis or death. Glucose-6-phosphate dehydrogenase (G6PD) levels and the results of the DAT were variable. In two of the three cases the hemolysis recurred with metformin rechallenge, which increases the likelihood of a causal relationship according to the Naranjo adverse drug reaction probability scale [4].

**Table 2:** Reported cases of metformin-induced hemolytic anemia

Case report	Patient's age (years)	Gender	Time from starting metformin to onset of symptoms	Direct Coombs	G6PD level	Recurrence of hemolysis with metformin rechallenge	Outcome
Lin et al. [1]	46	Male	10 days	'Equivocal'	Normal	Yes	Recovery
Kashyap and Kashyap [2]	51	Female	9 days	Positive (-IgG, +C3)	Normal	Yes	Recovery
Meir et al. [3]	68	Female	14 days	Negative	Decreased	N/A	Recovery
Packer et al. (this study)	56	Male	1 to 2 days	Positive (+IgG, -C3)	N/A	N/A	Death

Other possible causes of fulminant hemolysis must be considered in this patient. The lack of schistocytes makes micro-angiopathic processes such as thrombotic thrombocytopenic purpura (TTP) or disseminated intravascular coagulation less likely. In addition, the absence of fever and the patient's normal platelet count reduce the likelihood of TTP. There are several case reports of ciprofloxacin-induced hemolysis [5], but our patient had already developed anemia and hemoglobinuria suggestive of ongoing hemolysis before being started on ciprofloxacin in the emergency room. Another possibility is Evans syndrome, which is a DAT-positive hemolytic anemia and immune thrombocytopenia with no known underlying etiology. Evans syndrome causes chronic hemolytic anemia, which our patient did not have, and it is rarely fulminant. A third possibility is hemolytic anemia associated with acute hepatitis, most commonly hepatitis A, which has been described as a cause of fulminant and sometimes fatal hemolysis in patients with G6PD deficiency [6,7]. As hepatitis serologies and G6PD levels are not available for this patient, it is impossible to rule out acute hepatitis as the underlying cause of the hemolysis. However, at an office visit 4 days prior to admission, this patient had no prodromal signs or symptoms of hepatitis, and nothing in his travel or dietary history was suggestive of a risk for hepatitis A. In addition, hemolysis can cause a significant increase in AST and a more moderate increase in ALT owing to the release of these transaminases from the lysed RBCs [8]. As there is more AST than ALT in RBCs, a high AST-to-ALT ratio would be expected in a case of hemolysis-induced transaminitis. The presence a high AST-to-ALT ratio in this case suggests that massive hemolysis could account for all of the transaminase abnormalities.

The predictive value of a positive DAT for an immune etiology in a patient with hemolytic anemia is 83% [9]. DAT reactivity in our patient was 4+ to polyspecific, 4+ to IgG and negative to C3. The patient's serum reacted with all reagent RBCs tested by the indirect antiglobulin test. Adsorption of aliquots of serum with ZZAP-treated autologous RBCs failed to remove all reactivity (ZZAP is a papain and dithiothreitol reagent used to clear autoantibody from the patient's serum). Adsorption of aliquots of the patient's serum with R1R1, R2R2, and RBC samples revealed the presence of an autoantibody with 'e-like' specificity in the adsorbed serum. Serum antibody reactivity was consistent with a warm autoantibody (panagglutinin) and an antibody with 'e-like' specificity at anti-human globulin. These results could be consistent with either a warm autoimmune hemolytic anemia (WAIHA) or a drug-induced immune hemolytic anemia (DIIHA) with autoantibody formation (alpha-methyldopa type), which could explain the persistence of hemolysis in this case despite the discontinuation of metformin. Unlike DIIHAs involving neoantigen (immune complex) formation or drug adsorption onto the RBCs, hemolysis owing to drug-induced autoantibodies can persist for several days to months after the drug is stopped. The presence of an autoantibody directed to the Rh system ('e-like' specificity) can occur with WAIHA but is more commonly seen with DIIHA [10]. The strength of the DAT reactivity does not necessarily argue for a warm autoantibody; Joshua et al. have shown that the DAT is strongly positive (at least 2+) in 75% of patients with drug-associated hemolysis [11].

The possible mechanism of metformin-induced hemolytic anemia discussed here is different from that proposed by Kashyap and Kashyap in their report [2]. Their patient's DAT was positive for anti-C3 and negative for anti-IgG, which suggests the formation of an antibody against the erythrocyte-drug complex. In contrast, our patient's DAT was consistent with autoantibody formation. This is not necessarily a contradiction, since it has been shown that the same drug can cause many if not all of the mechanisms of DIIHA. In fact, one mechanism may simply be more pronounced and identifiable in a particular patient. Observations of DIIHA caused by third-generation cephalosporins support the notion of multiple mechanisms for the same drug [12].

Unfortunately, stored samples of this patient's blood are not available to test for drug-dependent antibodies. Ultimately, we cannot be certain whether this patient had WAIHA or drug-induced hemolysis caused by an autoantibody. If he did have WAIHA, we are unable to find any clear precipitating cause for his catastrophic hemolysis other than the initiation of metformin.

## Conclusion

This patient developed fulminant and fatal Coombs-positive hemolytic anemia that was temporally related to the initiation of metformin treatment, in the absence of any other likely cause. The Naranjo probability score is 3 ('possible') for a metformin drug reaction, but in view of the fatal outcome we think it is important to make clinicians aware of the possibility of rare but severe hemolysis with metformin treatment.

## Abbreviations

ALT: alanine aminotransferase; AST: aspartate aminotransferase; DAT: direct antiglobulin test; DIIHA: drug-induced immune hemolytic anemia; G6PD: glucose-6-phosphate dehydrogenase; RBC: red blood cell; TTP: thrombotic thrombocytopenic purpura; WAIHA: warm autoimmune hemolytic anemia.

## Competing interests

The authors declare that they have no competing interests.

## Authors' contributions

CDP researched the case, analyzed the laboratory data and was chief author of the manuscript. TRH assisted with the analysis of the data, helped substantially with the discussion and contributed to the manuscript. SAA cared for the patient, assisted with the details of the case report, helped with the discussion and assisted in revising the manuscript. All authors read and approved the final manuscript.

## Consent

Written informed consent was obtained from the patient's next-of-kin for publication of this case report and accompanying images. A copy of the written consent is available for review by the Editor-in-Chief of this journal.
